# Editorial: Metabolic dysregulation as a response to viral infections - a new culprit of metabolic diseases

**DOI:** 10.3389/fendo.2026.1991376

**Published:** 2026-09-16

**Authors:** Alpo Vuorio, Timo E. Strandberg, Petri T. Kovanen

**Affiliations:** 1 Department of Forensic Medicine, University of Helsinki, Helsinki, Finland; 2 Mehiläinen Aviapolis Health Centre, Vantaa, Finland; 3 Clinicum, University of Helsinki, Helsinki, Finland; 4 Helsing University Hospital, Helsinki, Finland; 5 University of Oulu, Oulu, Finland; 6 Wihuri Research Institute, Helsinki, Finland

**Keywords:** diabetes, hepatitis, immune signalling, metabolic dysregulation, pandemic, viral infections

It is part of the nature of viruses that when causing infections in humans they rewire metabolic routes like glycolysis and lipids synthesis and modulate immune signalling to secure their replication and assembly (1). This readjusting causes metabolic and immunological footprints influencing infections and contributing to the development or progression of metabolic diseases (2, 3). A global advanced understanding of this topic is necessary.

The role of viruses as modifiers of metabolic routes is increasing. Climate change has triggered ongoing changes in ecological systems, and it thereby increases the likelihood of zoonotic spillover events. This danger is further impacted by societal, demographical, and behavioural changes and thus it clearly increases the risk of a next pandemic (4–7). World Health Organization’s (WHO) efforts to strengthen pandemic preparedness are based on the development of the diagnostics and therapeutics of infectious diseases (8).

It is, however, easily forgotten that a bidirectional interaction between SARS-CoV-2 infection and metabolic dysfunction exists many years after pandemic (9). This means that any metabolic disease may worsen the prognosis of infections and that the SARS-CoV-2 infection may initiate or even cause the progress of metabolic diseases. Indeed, we are still paying a significant excess of costs related to the SARS-CoV-2 epidemic which triggered new metabolic diseases and increased the burden related to their continuous impact especially on vascular health (10–12).

The additional burden caused by the COVID-19 pandemic has been shown in several population groups. In a study involving almost 14 million individuals younger than 18 years in England, 28% had COVID-19 (13). The COVID-19 patients in this study had a higher risk of rare vascular and inflammatory diseases up to 12 months after the first COVID-19 diagnosis. Moreover, in a prospective multinational study which recruited 2390 individuals (aged 50 ± 15 yrs, 49%women), it was shown that COVID-19 survivors have long-term cardiovascular complications (14). Importantly, a multi-centre pilot cohort study revealed that COVID-19 contributes to persistent cardiometabolic risk after recovery (15), a result that confirmed the findings of an earlier larger study involving 5059 patients with non-severe SARS-CoV-2 infection (16). In a recent study of 100 adults with previous SARS-CoV-2 infection, changes in endothelial dysfunction and NO-metabolism were detectable at a median of 37 weeks after the infection (17). The immune activation and proinflammatory responses characterized by upregulation of JAK-STAT, interleukin-6, complement and T cell exhaustion pathways have been shown 180 days after the initial infection (18). Also, a mitochondrial bioenergetic collapse has been demonstrated to participate in the longer-term effects of SARS-CoV-2 infection (19). This has been recently explained by mitochondria- related DNA methylation which could then affect the expression of genes involved in the progression of the SARS-CoV-2 infection (20).

The majority of articles in this collection are related to the metabolic changes caused by SARS-CoV-2 infection. In a systematic review and meta-analysis, Lawal et al. studied metabolic changes at least three months after recovery from SARS-CoV-2 infection, and the authors found that COVID-19 may be associated with an increased risk of diabetes and with persistent metabolic dysregulation. Expectedly, in their review, Shitaye et al. found that in SARS-CoV-2 infection increased cytokine levels and dysregulation of the ACE2/renin-angiotensin system pathway together with dysregulation of the interferon regulatory factors appear to be among the mechanisms that are linked to multiple inter-organ pathologies and to metabolic alterations including new-onset diabetes. Moreover, the etiologic factors involved in the induction of a significant increase in insulin resistance and new-onset diabetes caused by SARS-CoV-2 were discussed in the review by Zhu et al. Surprisingly, in the retrospective clinical study of 682 diabetic patients having different stages of Cardiovascular-Kidney-Metabolic (CKM) syndrome stages, it was shown that post-SARS-Cov-2 progression was observed around 25% cases, but this progress was not driven by renin-angiotensin system inhibitors (Zhang et al.). Mechanistically, SARS-CoV-2 infection may cause failure of the compensatory mechanisms involved in the pancreatic β-cell function.

In the retrospective study of 688 patients with type 2 diabetes (T2D) with a history of SARS-CoV2 infection, overweight was found to accelerate the progression of proteinuria (Wu et al.). Since COVID-19 seems to aggravate diabetic complications, Zhao et al. highlighted in their review the urgent need to re-define the management strategies involving diabetes patients both during and after the pandemic. In a study of 60 COVID-19 patients, Sanhueza et al. showed that neutrophil extracellular trap formation was enhanced by insulin, a finding that called for post-viral control of glucose metabolism. Zhang et al. discussed in their review about a theoretical paradigm shift and suggested that there is a need to discuss co-dysregulation of immunity and metabolism regarding diabetes. These authors remind us in more detail that hyperglycaemia and lipotoxicity interact and cause chronic inflammation and immune dysfunction.

The pathogenesis of post-COVID syndrome (PCS) was discussed in two articles submitted to this article collection (Tasoula et al., Huhmar et al.). Tasoula et al. found in their analysis of transcriptomic, proteomic, and metabolomic datasets that mitochondrial dysfunction, suppressed oxidative phosphorylation, and immune dysregulation have an important impact on PCS. This finding links bioenergetic dysfunction with chronic immune activation. In a case series study, Huhmar et al. found that a combination of high serum and low urine osmolality is associated with poorer health status in PCS. Potentially, this finding offers an opportunity to use measurements of serum and urine osmolality as prognostic markers in PCS.

The bidirectional effects between infections and metabolic dysregulation resemble the multimetabolic dysregulation caused by infections. Islam et al. recall in their review article that there are several pathogens which have been shown to cause alterations in metabolism including *Helicobacter pylori, Staphylococcus aureus, Escherichia coli and* hepatitis viruses in addition to SARS-CoV-2 infection. The authors wish to see diabetes, which they use as an example in their review, not only as an endocrinological disorder but also as a systemic immunometabolic disease. So, it is important to see infections as modulators of metabolic homeostasis. A surprising finding related to siderophore pyoverdine (PVD) -induced iron dysregulation leading to avascular endothelial barrier dysfunction detected in Human Umbilical Vein Endothelial Cells was reported by Zhang et al. PVD impairs the endothelial barrier by activating oxidative stress which leads to downregulation of junctional proteins. Thus, it was shown in this study that PVD is a key mediator of vascular injury in diabetic foot infection. DFI is still a leading cause of morbidity and mortality in diabetic patients, with *Pseudomonas aeruginosa* serving as a predominant pathogen due to its arsenal of virulence factors.

Hepatitis has been shown to be associated with metabolic dysfunction in several studies (21, 22).

Behar-Lagares et al. characterized plasma lipidomic profiles of 58 patients having a hepatitis C (HCV) and HIV coinfection. It is known that 15-30% patients with HCV develop advanced chronic liver disease (23). In their study, Behar-Lagares et al. also found that especially phosphatidylcholines, phosphatidylethanolamines and triglycerides were significantly depleted in those patients having advanced chronic liver disease, and that these patients represented about 17% of the cohort. The authors conclude that dysregulation of hepatic lipid metabolism appears to play a critical role in the pathogenesis of hepatic decompensation. Hepatitis B virus (HBV) -related pathogenesis is discussed in the review by Tao et al. Authors of this review recall that HBV-associated metabolic dysfunction and the associated fatty liver disease increase the risk of hepatic malignancy. The findings may enable us to better identify high-risk individuals.

A case report by Cao et al. was selected to be part of the collection to remind the reader of the rare case of pheochromocytoma-induced severe diabetes. Although this rare type of induced diabetes was severe, it was reversible after early surgical intervention.

All in all, the COVID-19 pandemic has played an important role in increasing our awareness and understanding of how a severe viral infection may affect metabolic pathways. In fact, the SARS-CoV-2 pandemic has triggered cooperation between different groups of researchers, and it has also deepened the understanding between different scientific disciplines. However, there is still a considerable lack of understanding of several pathological mechanisms which alter the whole-body, organ-specific, and cell-specific metabolism during a viral infection. Understanding these pathological mechanisms is a key factor in creating preventive therapies, and it must be substantially supported because the probability of future pandemics is increasing.

## References

[1] Palmer CS . Innate metabolic responses against viral infections. Nat Metab. (2022) 4:1245–59. doi: 10.1038/s42255-022-00652-3 36266542

[2] Sumbria D Berber E Mathayan M Rouse BT . Virus infections and host metabolism-can we manage the interactions? Front Immunol. (2021) 11:594963. doi: 10.3389/fimmu.2020.594963 33613518 PMC7887310

[3] Li J Wang Y Deng H Li S Qiu HJ . Cellular metabolism hijacked by viruses for immunoevasion: potential antiviral targets. Front Immunol. (2023) 14:1228811. doi: 10.3389/fimmu.2023.1228811 37559723 PMC10409484

[4] Allen T Murray KA Zambrana-Torrelio C Morse SS Rondinini C Di Marco M . Global hotspots and correlates of emerging zoonotic diseases. Nat Commun. (2017) 8:1124. doi: 10.1038/s41467-017-00923-8 29066781 PMC5654761

[5] Radparvar S . From Black Death to COVID-19: Lessons learned from the past pandemics. Infez Med. (2025) 33:461–74. doi: 10.53854/liim-3304-12 41384157 PMC12695168

[6] Shi X Li T Wang Y Liu Q Wang J Ban J . Climate change and infectious diseases: translating evidence into action. Infect Dis Poverty. (2026) 15:66. doi: 10.1186/s40249-026-01468-z 42271449 PMC13251223

[7] Marani M Katul GG Pan WK Parolari AJ . Intensity and frequency of extreme novel epidemics. Proc Natl Acad Sci USA. (2021) 118:e2105482118. doi: 10.1073/pnas.2105482118 34426498 PMC8536331

[8] Van Kerkhove M . Preparing for the next pandemic: Learning from COVID-19 to build what comes next. Open Forum Infect Dis. (2026) 13:ofag348. doi: 10.1093/ofid/ofag348 42434385 PMC13350923

[9] Perakakis N Harb H Hale BG Varga Z Steenblock C Kanczkowski W . Mechanisms and clinical relevance of the bidirectional relationship of viral infections with metabolic diseases. Lancet Diabetes Endocrinol. (2023) 11:675–93. doi: 10.1016/S2213-8587(23)00154-7 37524103

[10] Kovanen PT Vuorio A . SARS-CoV-2 reinfection: Adding insult to dysfunctional endothelium in patients with atherosclerotic cardiovascular disease. Atheroscl Plus. (2023) 53:1–5. doi: 10.1016/j.athplu.2023.06.002 37293388 PMC10238112

[11] Spetz M Natt Och Dag Y Li H Nyberg F Rosvall M . Covid-19 and cardiovascular disease in a total population-study of long-term effects, social factors and Covid-19-vaccination. Nat Commun. (2025) 16:10115. doi: 10.1038/s41467-025-66270-1 41253781 PMC12627555

[12] Dalamaga M Karampela I Liu J . COVID-19 and metabolic health: Lessons from a global pandemic. Metabol Open. (2026) 29:100448. doi: 10.1016/j.metop.2026.100448 42005041 PMC13083650

[13] Sampri A Shi W Bolton T Ip S Knight R Walker V . Vascular and inflammatory diseases after COVID-19 infection and vaccination in children and young people in England: a retrospective, population-based cohort study using linked electronic health records. Lancet Child Adolesc Health. (2025) 9:837–47. doi: 10.1016/S2352-4642(25)00247-0 41198364

[14] Bruno RM Badhwar S Abid L Agharazii M Anastasio F Bellien J . Accelerated vascular ageing after COVID-19 infection: the CARTESIAN study. Eur Heart J. (2025) 46:3905–18. doi: 10.1093/eurheartj/ehaf430 40819656 PMC12517754

[15] Mohamed F Gunter S Currin S Raal FJ Kalla IS Millen AME . Metabolic implications of COVID-19 with a focus on lipid profiles and inflammatory markers. Sci Rep. (2026) 16:6217. doi: 10.1038/s41598-026-37439-5 41582261 PMC12905385

[16] Washirasaksiri C Sayabovorn N Ariyakunaphan P Kositamongkol C Chaisathaphol T Sitasuwan T . Long-term multiple metabolic abnormalities among healthy and high-risk people following non-severe COVID-19. Sci Rep. (2023) 13:14336. doi: 10.1038/s41598-023-41523-5 37653091 PMC10471587

[17] Oestreich M Schmidt M Dittrich J Baber R Gaudl A Reinicke M . Endothelial dysfunction and metabolic biomarkers in post-COVID-19 syndrome. Sci Rep. (2026) 16:15067. doi: 10.1038/s41598-026-50965-6 42129413 PMC13172428

[18] Aid M Boero-Teyssier V McMahan K Dang R Doyle M Belabbaci N . Long COVID involves activation of proinflammatory and immune exhaustion pathways. Nat Immunol. (2026) 27:61–71. doi: 10.1038/s41590-025-02353-x 41388153 PMC12764429

[19] Chen TH Jeng TH Lee MY Wang HC Tsai KF Chou CK . Viral mitochondriopathy in COVID-19. Redox Biol. (2025) 85:103766. doi: 10.1016/j.redox.2025.103766 40680383 PMC12284291

[20] Kumari D Singh S Chauhan D Dange K Ganju L Dogra V . SARS-CoV-2 altered mitochondrial DNA methylation in Indian COVID-19 patients. Sci Rep. (2026) 16:2405. doi: 10.1038/s41598-025-28945-z 41554758 PMC12820342

[21] Nastasa R Stanciu C Stratina E Zenovia S Stafie R Rotaru A . Screening of advanced fibrosis and metabolic dysfunction-associated steatotic liver disease using transient elastography in vulnerable population. Sci Rep. (2025) 15:33028. doi: 10.1038/s41598-025-18733-0 41006460 PMC12475056

[22] Yan Y Wei Z Zheng M Lu M Wang X . HBV and host metabolic crosstalk: Reprogramming pathways for viral replication and pathogenesis. Virol Sin. (2025) 40:685–93. doi: 10.1016/j.virs.2025.09.008 41022265 PMC12665413

[23] Ferreira J Caldeira J Bicho M Faustino P Serejo F . Hepatitis C virus: An overview of its chronic impact on liver function, metabolic dysregulation, inflammatory-oxidative ppthogenesis and epigenetic memory. Int J Mol Sci. (2026) 27:3559. doi: 10.3390/ijms27083559 42074199 PMC13116419

